# GOAT: Gene-level biomarker discovery from multi-Omics data using graph ATtention neural network for eosinophilic asthma subtype

**DOI:** 10.1093/bioinformatics/btad582

**Published:** 2023-09-22

**Authors:** Dabin Jeong, Bonil Koo, Minsik Oh, Tae-Bum Kim, Sun Kim

**Affiliations:** Interdisciplinary Program in Bioinformatics, Seoul National University, Seoul 08826, Republic of Korea; Interdisciplinary Program in Bioinformatics, Seoul National University, Seoul 08826, Republic of Korea; AIGENDRUG Co., Ltd, Seoul 08826, Republic of Korea; School of Software Convergence, Myongji University, Seoul 03674, Republic of Korea; Department of Allergy and Clinical Immunology, Asan Medical Center, University of Ulsan College of Medicine, Seoul 05505, Republic of Korea; Interdisciplinary Program in Bioinformatics, Seoul National University, Seoul 08826, Republic of Korea; AIGENDRUG Co., Ltd, Seoul 08826, Republic of Korea; Department of Computer Science and Engineering, Seoul National University, Seoul 08826, Republic of Korea; Interdisciplinary Program in Artificial Intelligence,, Seoul National University, Seoul 08826, Republic of Korea

## Abstract

**Motivation:**

Asthma is a heterogeneous disease where various subtypes are established and molecular biomarkers of the subtypes are yet to be discovered. Recent availability of multi-omics data paved a way to discover molecular biomarkers for the subtypes. However, multi-omics biomarker discovery is challenging because of the complex interplay between different omics layers.

**Results:**

We propose a deep attention model named Gene-level biomarker discovery from multi-Omics data using graph ATtention neural network (GOAT) for identifying molecular biomarkers for eosinophilic asthma subtypes with multi-omics data. GOAT identifies genes that discriminate subtypes using a graph neural network by modeling complex interactions among genes as the attention mechanism in the deep learning model. In experiments with multi-omics profiles of the COREA (Cohort for Reality and Evolution of Adult Asthma in Korea) asthma cohort of 300 patients, GOAT outperforms existing models and suggests interpretable biological mechanisms underlying asthma subtypes. Importantly, GOAT identified genes that are distinct only in terms of relationship with other genes through attention. To better understand the role of biomarkers, we further investigated two transcription factors, *CTNNB1* and *JUN*, captured by GOAT. We were successful in showing the role of the transcription factors in eosinophilic asthma pathophysiology in a network propagation and transcriptional network analysis, which were not distinct in terms of gene expression level differences.

**Availability and implementation:**

Source code is available https://github.com/DabinJeong/Multi-omics_biomarker. The preprocessed data underlying this article is accessible in data folder of the github repository. Raw data are available in Multi-Omics Platform at http://203.252.206.90:5566/, and it can be accessible when requested.

## 1 Introduction

Asthma is a chronic inflammatory disorder of the airways of the lungs characterized by a clinical syndrome of bronchial hyper-responsiveness and reversible/irreversible airflow obstruction, which is a heterogeneous disease in terms of clinical manifestation and treatment responsiveness. Heterogeneity is due to the multi-factor nature of asthma pathophysiology: polygenecity, multiple pathways, and gene–environment interactions (Tang *et al.* 2020). Characterization of asthma subtypes according to demographic/pathophysiological properties has emerged to resolve the heterogeneity in asthma (Kuruvilla *et al.* 2019).

While demographic/pathophysiological properties have long been key features for asthma subtyping, recent molecular profiling from different omics technologies is an evolving paradigm for dissecting heterogeneity and identifying etiology/pathophysiology—for example, a microarray study in bronchial airway identified *IL13*-inducible genes showed differential transcriptional expression in T2-high/T2-low subtypes (Woodruff *et al.* 2009). Although various asthma subtypes are established, molecular biomarkers to explain pathophysiology of the subtypes’ stratification are yet to be discovered.

Several studies have been proposed to identify molecular biomarkers for asthma subtypes, but those studies are restricted to the single-omics analysis (Kuo *et al.* 2017, Quoc *et al.* 2021). Multi-omics technology can comprehensively analyze the molecular profiling of patients, thus it unravels the pathophysiology of asthma subtypes in which the complex interplay of biomolecules is involved (Ivanova *et al.* 2019).

Cohort for Reality and Evolution of Adult Asthma in Korea (COREA) is a Korea nationwide, observational, and long-term cohort study of adult asthma that unprecedentedly covers a full spectrum of asthma phenotypes with multi-omics profiling of 1260 asthma patients since 2005 (Kim *et al.* 2009). With the availability of this multi-omics data, the goal of our study is to develop models for discovering multi-omics biomarkers for asthma subtypes.

Biomarker is an indicator whose observed measure serves as a medical sign of patient status, e.g. a particular subtype of asthma. Among various biomolecules from multiple modalities (e.g. DNA loci, methylated nucleobases, metabolites, mRNA, or proteins), discovery of gene-level biomarkers is common and of broader interest as each biomolecule can be interpreted as a regulatory status or a product of a gene. Since discriminating patient status is regarded as a classification task, the gene-level biomarker discovery problem is formulated as a feature selection problem to enumerate discriminative features or genes for the task, which is a supervised approach that discovers predictive biomarkers of disease.

### 1.1 Related works: existing biomarker discovery methods

#### 1.1.1 Single-omics biomarker discovery

Statistical testing is a conventional method of discovering biomarkers by analyzing omics profiles, which usually identifies genes that show differential abundance between phenotypes (Woodruff *et al.* 2009, Ye *et al.* 2019)—for example differentially expressed gene (DEG) analysis of transcriptome data. Since there is emerging evidence that the genes underlying the disease tend to interact, several studies have been proposed to provide a set of interacting genes as biomarkers (Han *et al.* 2019, Chu *et al.* 2022).

#### 1.1.2 Multi-omics biomarker discovery

To handle the multi-modality and heterogeneity of multi-omics data, several multi-omics data integration methods have been proposed. One strategy for multi-omics integration is unsupervised methods that extract biological insights without phenotypic information of samples. Some methods use networks of sample as a basis for integration, which constructs a sample similarity network of each omics then yields a converged sample network across omics via network fusion (Wang *et al.* 2014) or via random walk with restart (RWR) (Wen *et al.* 2021). Although these methods were successful for patient stratification, its applicability to biomarker discovery is limited because the methods produce relative relations between patients; thus, they are not designed to extract important features in classifying patients. Another line of works is to convert multi-omics data into common feature space, yielding latent representations of samples. These include matrix factorization (Zhang *et al.* 2012), sparse generalized canonical correlation analysis (sGCCA) (Tenenhaus *et al.* 2014), multi-omics factor analysis (MOFA) (Argelaguet *et al.* 2018). Although these methods were not designed for multi-omics biomarker discovery, representation of patients can be fed into the classification model so that they are applicable to biomarker discovery.

Recently, another strategy of supervised methods that use phenotypic information of samples has been developed, which is multi-omics integration methods specified for biomarker discovery. Notable advances in multi-omics biomarker discovery take the relations between omics layers into account. For example, iDRW (Kim *et al.* 2021) constructs a pathway-level representation of patients from multi-omics data via RWR on multiplex gene–gene graph, so that classifiers discriminate between patients with pathway-level representations and identify important pathways. Data Integration Analysis for Biomarker discovery using Latent cOmponents (DIABLO) (Singh *et al.* 2019) discovers biomarkers by integrating multi-omics data with sGCCA, which captures the common biological variation between multi-omics by maximizing the correlation between omics. Although these methods take into account relations among omics, these models are not designed to identify gene-level biomarkers considering complex interaction among biomolecules. Multi-Omics Graph cOnvolutional NETworks (MOGONET) (Wang *et al.* 2021) is one of the pioneering deep learning models that learn omics-specific features from omics-specific patient similarity matrices and integrates multi-omics through a view correlation discovery network to explore the latent cross-omics correlations. Since the multi-omics integration is conducted at patient level, the interplay of omics features is neglected.

### 1.2 Challenges

The major challenges for gene-level biomarker discovery are as follows:

- There are many DEGs, typically several hundreds to even thousands. Given this large number of DEGs, it is difficult to identify gene-level biomarkers even with statistical information, e.g. *P*-val.
- Use of multi-omics data can help identify gene-level biomarkers effectively. However, complexity of relationship becomes very high. It is well-known that genes function through complex interactions. Considering complex relationships in multi-omics data for gene regulation or gene products makes biomarker discovery extremely challenging.

To address these challenges, the use of sophisticated network analysis and technologies is necessary.

### 1.3 Our approach

We propose a novel deep graph attention model for biomarker discovery from asthma multi-omics data. Our approach is distinct from existing methods in that our deep graph learning model is combined with network propagation that uses explicit molecular interaction among biomolecules.

Our main idea is (i) to prioritize genes through network propagation that globally simulates all possible interactions of genes in a network, which can hardly be discovered via independent statistical hypothesis testing of genes and (ii) to identify genes that model complex interaction of multi-omics biomolecules with graph attention neural network. In other words, we aim to identify genes that reflect multi-omics regulation of genes whose impact can be captured in terms of relationship with other genes.

The main contributions of our work are as follows,

- We propose a novel graph deep learning model for biomarker discovery that incorporates multi-profile data with gene–gene interaction network.
- We performed analysis of multi-omics data for physiological/clinical subtype criteria from COREA asthma cohort, which covers unprecedentedly a full spectrum of asthma phenotypes with multi-omics profiling, and discovered biomarkers for eosinophilic asthma.

## 2 Materials and methods

### 2.1 Overview of GOAT

**Figure 1.**
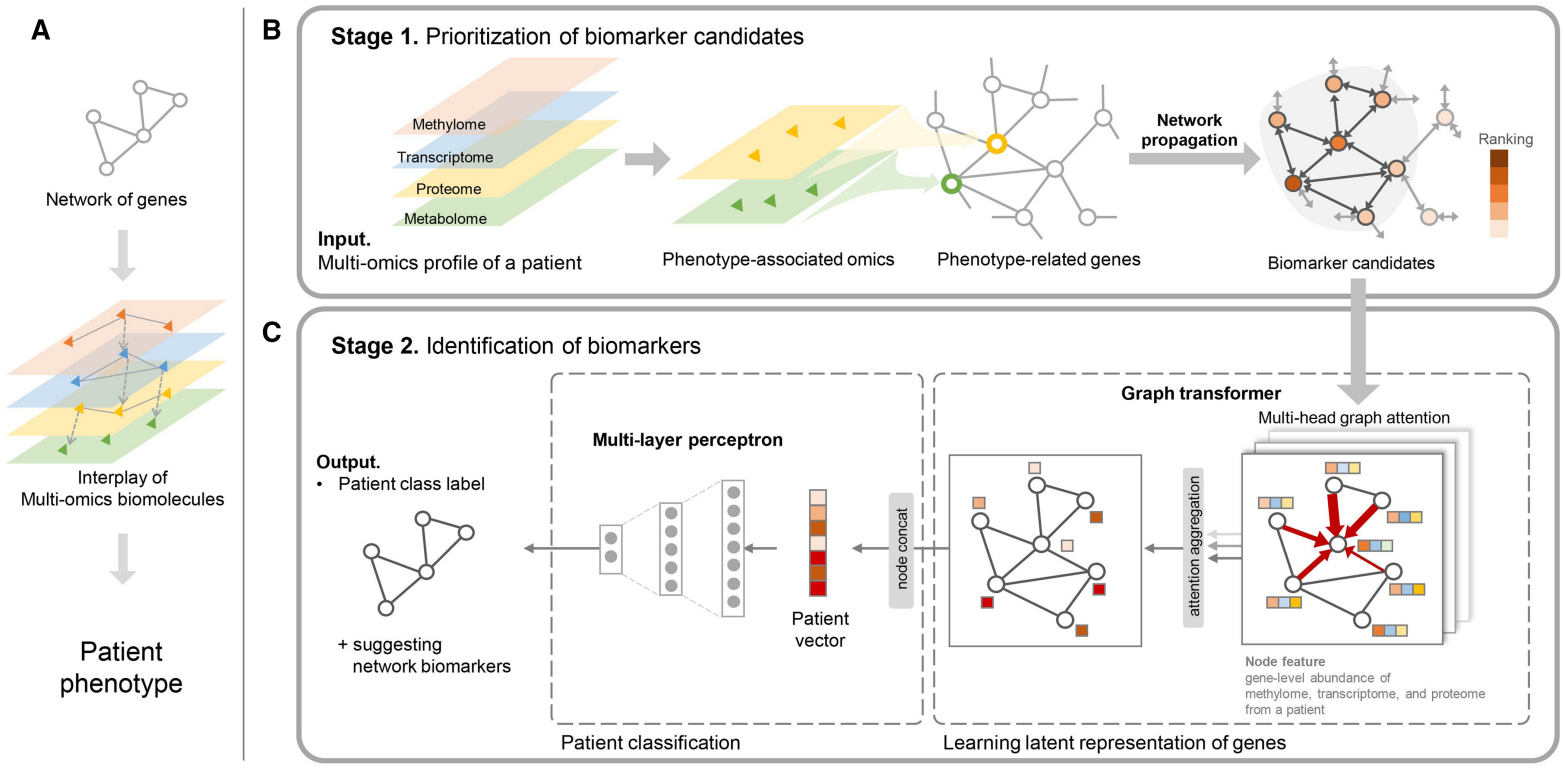
Schematic overview of GOAT. (A) Patient phenotype is the result of gene interactions regulating the interplay of multi-omics biomolecules. Our goal is to discover a network of genes that explains patient phenotype from multi-omics data. GOAT consists of two stages: prioritization of biomarker candidates using network propagation and identification of biomarkers using attention in graph transformer. (B) In the 1st stage, we prioritized genes that are important in discriminating phenotypes from phenotype-associated omics, proteome, and metabolome. Significant proteins and metabolites are identified and mapped to a gene-gene interaction graph. The triangle denotes the significant protein/metabolite features and the bold circle denotes the genes related to the significant features. Then network propagation prioritizes biomarker candidates. (C) In the 2nd stage, the protein–protein interaction network is trimmed with the genes prioritized in the 1st stage to make a network of biomarker candidates. Then the gene-level quantity of gene is given as node features (boxes next to the nodes) to generate graph instances of each patient. Multi-head graph attention from graph transformer model is trained to learn the latent representation of genes and the representations are concatenated to generate a patient vector. Lastly, multi-layer perception conducts a graph classification task, yielding genes with high attention weights as network biomarkers.

We propose a biomarker identification model from heterogeneous multi-omics data (Fig. 1). The core of this method consists of two stages: (1) prioritization of biomarker candidates using network propagation, and (2) identification of biomarkers using attention weights from an interpretable graph neural network (GNN).

The rationales underpinned in the approach are that (i) biomarkers are disease-related genes and (ii) biomarkers are the genes that show prediction capabilities on unknown patients. The first stage can be interpreted as a ‘feature selection step’ that prioritizes genes according to the relevance of disease subtypes where the high-ranked genes are regarded as biomarker candidates. Then, the second stage identifies biomarkers that show predictive power in discriminating subtypes, which is a ‘classification step’. The model identified potential biomarker genes and a subnetwork centered around the biomarker genes as output, which delineates the underlying biological processes that differentiate phenotypes.

### 2.2 Stage 1. Network propagation for prioritization of biomarker candidates

Network propagation is a network analysis technique that prioritizes nodes in a network by iteratively diffusing the resources or influence of seed nodes along the network edges, simultaneously considering all relations between nodes (Cowen *et al.* 2017). As a result, the diffused resources reflect the effect of seed nodes. Due to its properties, network propagation has been widely used in disease gene prioritization in the field of bioinformatics (Sun *et al.* 2019). Here we used network propagation to compute the possibility of each gene being a biomarker.

Which genes should be selected as seeds that are the core genes relevant to the disease? As proteome/metabolome is considered the most proximal omics layer to the phenome (Hasin *et al.* 2017, Jendoubi 2021), differentially abundant metabolites (DAMs) and differentially expressed proteins (DEPs) for each subtype are selected as seeds as described in the Supplementary method B. Since our goal is to prioritize gene-level biomarkers, we identified DAM-related genes (DAMgenes) that account for the metabolic difference from DAMs, using metPropagate (Graham Linck *et al.* 2020) which is a metabolic gene prioritization method that utilizes a gene-metabolite network. Top *k* genes with the highest ‘metPropagate’ score are regarded as DAMgenes.

The biomarker scores of genes are computed with a modified RWR (Bang *et al.* 2023) as follows,


$$p_{t}=(1-\beta )[(1-\alpha )W_{i}p_{t-1}+\alpha p_{0}]+\beta W_{f}p_{t-1},$$

where $p_{t}\in R^{d}$ is the node resources of the whole gene set in a network after $t^{\text{th}}$ iteration and *d* is the number of genes in a network. $p_{0}\in R^{d}$ is a binary vector indicating initial resources of a node where 1 means that the gene is a seed and 0 otherwise.

$W_{i}$ is the adjacency matrix of a gene–gene interaction network consisting of whole genes and $W_{f}$ is the normalized weight adjacency matrix of a gene–gene functional similarity network (Supplementary method C). α∈[0,1] is the probability of retaining the resources of seeds and β∈[0,1] is the probability of using the functional similarity network. The propagation process iterates until a convergence condition, $||p_{t+1}-p_{t}|{|}_{1}\leq 10^{-6}$.

After RWR converges, genes with the highest $k^{\prime }$ biomarker scores are regarded as biomarker candidates for each subtype. The union of subtype-specific biomarker candidates, *m* genes, is fed into the biomarker identification model in the following section.

### 2.3 Stage 2. Graph neural network for biomarker identification

A patient can be represented as a gene–gene interaction network since the pathophysiological status of a patient is the result of the cooperative regulation of multiple genes (Roy *et al.* 2014). To identify biomarkers that have predictive power in discriminating subtypes of asthma patients, we formulate the biomarker discovery problem as a graph classification task.

The goal is to learn a function f:G→L, where G is a set of patient graphs and *L* is the set of subtype labels, given a set of attributed patient graphs $D=\{(G_{1},l_{1}),(G_{2},l_{2}),\ldots ,(G_{n},l_{n})\}$, where $G_{i}$ and $l_{i}\in \{0,1\}$ denotes graph and subtype label of patient *i*, respectively. To model the function *f*, we utilized GNN on gene–gene interaction network. $G_{i}=(A_{G_{i}},X_{G_{i}})$ comprised a weighted adjacency matrix $A_{G_{i}}$ and an attribute matrix $X_{G_{i}}$. Topology of $A_{G_{i}}\in R^{m\times m}$ is processed by screening gene–gene interaction network with *m* biomarker candidates from the stage 1. $X_{G_{i}}\in R^{m\times n}$ represents a matrix of *m* genes and n=3 attributes, where the attribute corresponds to transcriptome, methylome, and proteome measurements at gene level.

The biomarker identification model consists of two deep learning modules: graph transformer and multi-layer perceptron (MLP). The graph transformer extracts latent gene representations from multi-omics data, which are then used by an MLP to perform patient classification based on the derived gene representations.

#### 2.3.1 Graph transformer for learning latent representation of genes

The goal is to learn a latent representation of genes from multi-omics data. One of the challenges is the complex interactions among genes. There exists a renowned publicly available gene–gene interaction network, protein–protein interaction (PPI) network, but PPI consists of interactions from multiple sources—experimentally validated physical interaction, co-expression, and text mining. Thus, interactions from PPI incorporate other omics information—for example, transcriptome–proteome level interactions. Therefore, two major challenges need to be tackled in exploiting the network: (i) all inter-omics/intra-omics relations are intermingled in a single network and (ii) ambiguity in defining inter-omics regulatory relations. Thus, we adopt a graph deep learning framework to model complex relationships between genes, using PPI network as a guide.

Graph transformer (Shi *et al.* 2021) is a powerful deep learning model to learn latent node representations based on attentive message passing along network edges. Attention layers in the model allow the model to pay attention to only certain parts of the input that are relevant to perform the task (Chaudhari *et al.* 2021). Multi-head attention mechanism, where several attention layers are stacked in parallel, enables learning of different representations of the input features. Not only it can model complex relations of nodes in a graph, its attentive message passing also allows models to highlight decisive nodes in a task.

In the context of our study, attention weights are learned for gene *i* in a network and its neighbor gene *j* and it can be interpreted as the probability of how much impact the gene *j* has in learning representation of gene *i*.

Since an attention head projects the initial gene features into a new subspace, multi-head attention enables the projection of genes into multiple different subspaces so that it is possible to learn the holistic gene representations through various perspectives.

In terms of implementation, each layer in graph transformer consists of multi-head attention layer and attention aggregation layer. The attention operation can be written as below:


$$\begin{array}{c} \alpha_{c,ij}^{\left(l\right)}=\frac{<q_{c,i}^{\left(l\right)},k_{c,j}^{\left(l\right)}>}{\sum_{u\in N(i)}<q_{c,i}^{\left(l\right)},k_{c,u}^{\left(l\right)}>}\\ q_{c,i}^{\left(l\right)}=W_{c,q}^{\left(l\right)}d_{i}^{\left(l\right)}+b_{c,q}^{\left(l\right)}\\ k_{c,j}^{\left(l\right)}=W_{c,k}^{\left(l\right)}d_{j}^{\left(l\right)}+b_{c,k}^{\left(l\right)}\\ v_{c,j}^{\left(l\right)}=W_{c,v}^{\left(l\right)}d_{j}^{\left(l\right)}+b_{c,v}^{\left(l\right)} \end{array},$$

where $\alpha_{c,ij}$ stands for attention weight of $l^{th}$ layer for each *c*-th head, $<q,k>=\text{exp}(\frac{q^{T}k}{\sqrt{d}})$ and *d* is the size of representation. The $W_{c,q}$, $W_{c,k}$, $W_{c,v}$, $b_{c,q}$, $b_{c,k}$, $b_{c,v}$ are trainable parameters and subscript *q*, *k*, *v* indicate query, key, and value, respectively. The feature attribute vectors $d_{i}$ and $d_{j}$ of nodes *i* and *j* are transformed into the query representation $q_{c,i}$ and the key representation $k_{c,j}$ to compute attention weight $\alpha_{ij}$.

After attention coefficients are computed, message aggregation from node *j* to node *i* across *C* head attention is denoted as follows,

$$d_{i}^{\left(l+1\right)}=|{|}_{c=1}^{C}[\sum_{j\in N(i)}\alpha_{c,ij}^{\left(l\right)}v_{c,j}^{\left(l\right)}],$$

where ‖ is an attention aggregation operation that concatenates multi-head attentions.

#### 2.3.2 Multi-layer perceptron for classification

After learning latent representations of genes from graph transformer, we concatenate gene representations to make a representation of a patient graph to predict subtype labels. The concatenated node representations are fed into MLP, and loss is calculated as binary cross entropy:


$$\text{loss}(y,\hat{y})=-\frac{1}{n}\sum_{i}^{n}[y_{i}\;\text{log}\;\hat{y_{i}}+(1-y_{i})\;\text{log}(1-\hat{y_{i}})],$$

where $y_{i}$ denotes for the true label and $\hat{y_{i}}$ is the predicted label for the patient graph *i*.

#### 2.3.3 Biomarker identification with attention weights

The most relevant gene interactions for discriminating subtypes are identified according to the attention weight matrix of genes. Larger the attention weight is, more important the pair of genes in discriminating subtypes. As the transformer layers compute the relative attention weights of the neighbor genes for each gene, we multiplied the attention weights by the degree of the anchor nodes to retrieve global patterns of attention weights across gene pairs. Finally, if the attention weight of a gene pair is greater than 1, genes in the gene pairs were considered as biomarkers. Since multiple pairs of genes can be selected, a union of the genes from the gene pairs are selected as biomarkers and a gene–gene interaction graph filtered by the biomarkers is suggested as a network biomarker.

## 3 Results

We analyzed 300 asthma patient data from COREA cohort that included contemporaneous multi-omics profiles and multiple physiological/clinical subtype labels. Four types of omics—methylome (22 255 genes), transcriptome (28 268 genes), proteome (341 proteins), and metabolome (407 metabolites)—are available. For more details on data processing, see Supplementary method D. From the subtypes defined according to the 16 phenotypic or endotypic features in the cohort (Supplementary Table S1), eosinophilic asthma (EA) and non-eosinophilic asthma (NEA) is the only subtype that can be discriminable with multi-omics data (Supplementary Fig. S1). The framework we develop will be applicable to the general multi-omics biomarker discovery problem, but for the asthma subtypes, biomarker discovery for EA/NEA subtypes was explored.

The validity of biomarkers was evaluated as the prediction performance in subtype classification for unseen data. To evaluate the generalized predictive power of GOAT, we conducted 10-fold cross-validation (CV) scheme where each CV fold was used as a held-out test set, while the samples from the other CV folds were used as a training set for biomarker prioritization and identification. For more details on the cross-validation scheme and hyperparameter tuning, see Supplementary method E and Supplementary Fig. S9. Prediction performance was measured as the area under the receiver operating characteristic curve (AUROC) and the area under the precision–recall curve (AUPRC).

### 3.1 Performance comparison with existing methods

**Figure 2.**
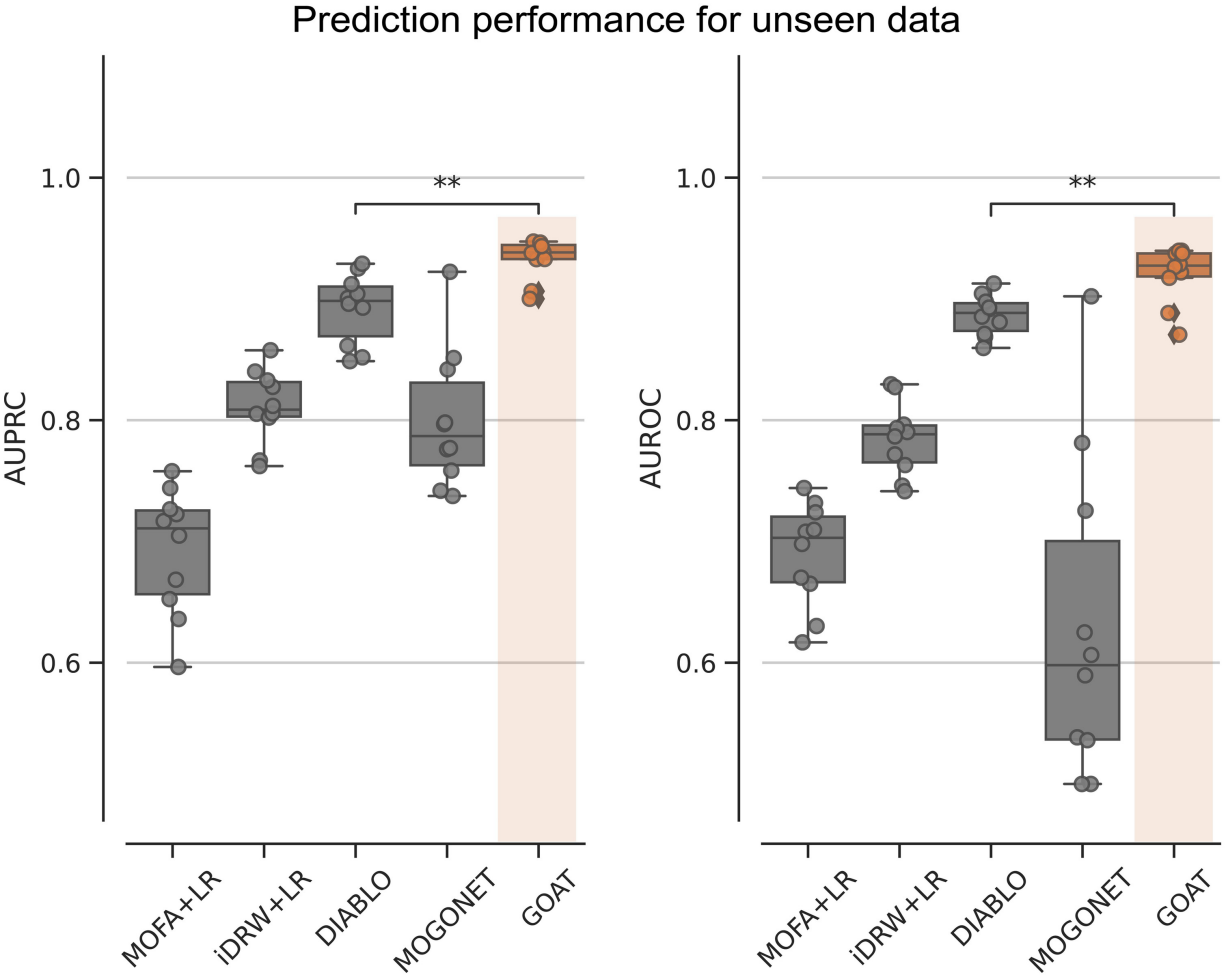
Performance comparison with existing methods. Each dot in the plots depicts test AUPRC/AUROC over 10-fold CV. Boxplot comparing GOAT (orange) and existing multi-omics biomarker discovery methods (grey). The center line denotes the median, the upper and lower box boundaries denote upper and lower quartiles, and the whiskers denote 1.5× interquartile range. Denoted statistical annotations are retrieved from *t*-test (**P <* *.01). AUPRC, area under the precision–recall curve; AUROC, area under the receiver operating characteristic curve; CV, cross-validation; LR, logistic regression.

To train multi-omics biomarker tools in comparison, we set the optimal hyperparameters that were previously identified for each model. We used logistic regression as a classifier to measure the predictive performance of biomarkers. Compared to existing multi-omics biomarker discovery tools—MOFA, iDRW, DIABLO, MOGONET, GOAT on average outperforms in subtype prediction of unseen test samples for any train-test set splits (Fig. 2).

### 3.2 Power of using multi-omics data for biomarker discovery

We replaced the feature selection method and classification model used in (1) the biomarker prioritization stage and in (2) the biomarker identification stage, respectively. We replaced our feature selection method, multi-omics network propagation (multi-omics NP), with other statistical feature selection methods from single-omics data—proteome and transcriptome.

**Figure 3.**
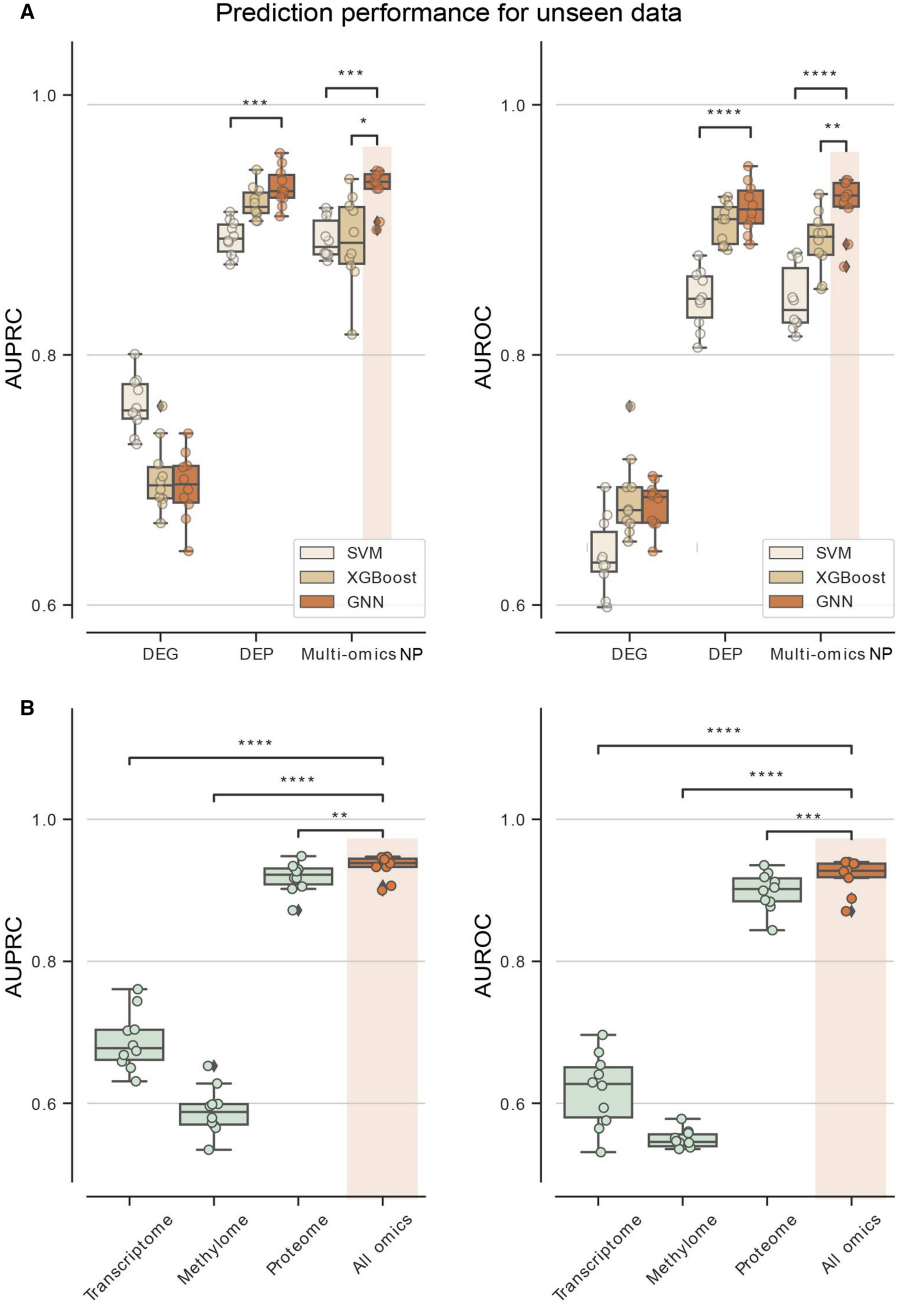
Power of using multi-omics network for biomarker discovery. Each dot in the plots depicts test AUPRC/AUROC over 10-fold CV. (A) Boxplot comparing feature selection method: DEG, DEP, and multi-omics NP. (B) Boxplot comparing the performance using multi-omics features (orange) and using single-omics features (green) in GNN model. When using single-omics features, only features from the specified omics were fed into the model. The center line denotes the median, the upper and lower box boundaries denote upper and lower quartiles, and the whiskers denote 1.5× interquartile range. Denoted statistical annotations are retrieved from *t*-test (*P < .05, **P < .01, ***P <* *.001, ****P < .0001). AUPRC, area under the precision–recall curve; AUROC, area under the receiver operating characteristic curve; CV, cross-validation; DEG, differentially expressed gene; DEP, differentially expressed protein; multi-omics NP, multi-omics network propagation; GNN, graph neural network.

We found that multi-omics NP showed significantly higher prediction performance (AUPRC: 0.94, AUROC: 0.93) compared to the feature selection via DEG analysis (AUPRC: 0.70, AUROC: 0.69), demonstrating the effectiveness of our biomarker candidate gene selection approach (Fig. 3A and Supplementary Fig. S3). This result is consistent with our experience of utilizing network propagation techniques. For example, in a study for anti-senescence activity of adipocytes (Lee *et al.* 2022), *SREBP1c* can hardly be detected by the DEG analysis (fold change only 1.07), but *SREBP1c* was ranked top 5 after aggregating gene interactions by network propagation. Our approach of modeling multi-omics data on a refined biological network using network propagation has resulted in a significant improvement in subtype prediction performance at the gene level. This suggests that our approach is effective in analyzing complex biological networks and identifying relevant features for subtype prediction.

GOAT also outperformed the DEP-based subtype classification (AUPRC: 0.93, AUROC: 0.92). Combined with variations in performances, the performance of GOAT was better than the performance of the DEP-based subtype classification statistically with *F*-test *P* value .101 for AUPRC and .103 for AUROC, respectively. Although the DEP-based subtype classification performed very well, even closer to the performance of our multi-omics method, note that our goal is to discover biomarkers rather than subtype classification. Identifying biomarkers from DEPs is not trivial for the following reasons. First, there are many DEPs, 133 DEPs for EA subtypes. Second, transcription factor (TF) biomarkers can hardly be detected by differential expression analysis. Third, since the roles of TFs cannot be easily detected, biological mechanisms underlying different subtypes can hardly be explained.

Additionally, a comparison of the model performance using single-omics features each versus all omics features, demonstrates that prediction performance using multi-omics attributes showed the highest prediction performance compared to the result of single-omics attributes in GNN model (Fig. 3B). Thus, the result suggests that utilizing multi-omics data is effective for biomarker discovery.

### 3.3 Discovery of mediator genes that can hardly be discovered by single-omics analysis

As a follow-up exploration of the biomarkers discovered, we analyzed the network of biomarkers to provide explanations for EA/NEA pathophysiology. We found that there exist 23 genes discovered solely by GOAT, not discovered in single-omics analysis of transcriptome, proteome, and metabolome data (Supplementary Fig. S4). Those genes include TFs: Catenin Beta 1 (*CTNNB1*) and Jun Proto-Oncogene (*JUN*) which is a subunit of AP-1 TF complex. *CTNNB1* and *JUN* are the TFs that are reported as significant TFs in TF enrichment analysis of the discovered biomarkers (Supplementary Table S4) connoting that the TFs are responsible for regulating the biomarkers.

**Figure 4.**
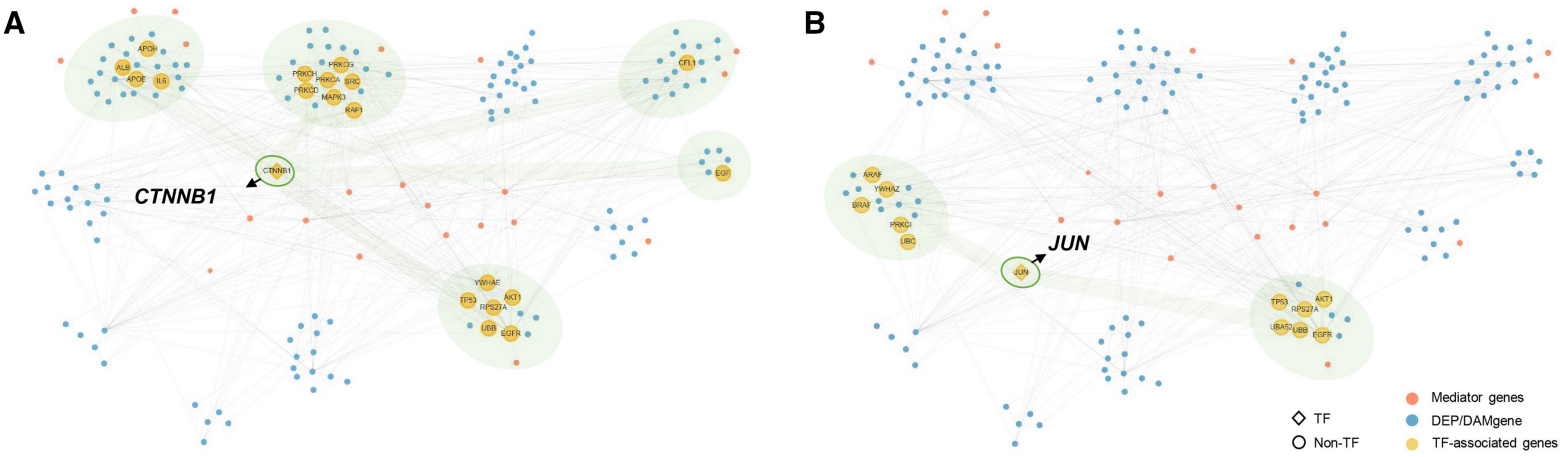
Subnetwork contains mediator genes that relay functional gene modules. Network of biomarkers discovered by GOAT. Each node in a network denotes a gene and the edge is retrieved from the gene-interaction network in GOAT. Rhombus indicates TFs while the circle indicates non-TFs. Blue nodes are the functional genes that are discovered by proteome/metabolome analysis, denoted as DEP/DAMgenes. Orange nodes are the genes that connect the functional gene modules, denoted as mediator genes. Yellow nodes are the genes connected to the selected TFs, (A) *CTNNB1* and (B) *JUN*, respectively. Green shade indicates functional modules connected to the TFs whose biological function is related to apoptotic process regulation. TF, transcription factor; DEP, differentially expressed protein; DAMgene, differentially abundant metabolite (DAM)-related gene.

We then asked whether the biomarkers discovered solely by GOAT have explanatory power for subtype pathophysiology. To better understand the biological meaning of biomarkers, we analyzed the biomarkers discovered at a network level (Fig. 4). Based on the gene–gene interaction network of our biomarkers, DEPs or DAMgenes are clustered to detect functional gene modules. Enriched gene ontology biological process (GOBP) terms of each module were mainly lipid/lipoprotein metabolic process, FC-γ signaling, and protein localization where there is no consistency of GOBPs across gene modules (Supplementary Fig. S5). However, negative regulation of apoptotic process emerges as a significant GOBP term after relaying gene modules connected via *CTNNB1* (Fig. 4A and Supplementary Table S2). *CTNNB1* is a gene that engages in WNT/β-catenin signaling that regulates cell fate and apoptosis whose role in eosinophil accumulation in EA asthma via negative regulation of apoptosis was reported by several existing studies (Kwak *et al.* 2015, Koopmans and Gosens, 2018). Consistent results are observed in the case of *JUN* (Fig. 4B and Supplementary Fig. S6 and Supplementary Table S3) whose role in mediating eosinophil apoptosis was reported (Hasala *et al.* 2007), thus it implies the possibility of modulating eosinophil accumulation via regulating apoptosis.

### 3.4 Distinct activity pattern of the regulatory biomarkers in EA versus NEA

Given that *CTNNB1* and *JUN* are discovered as regulatory TFs, we further investigated whether the transcription activities of *CTNNB1* and *JUN* differ between EA/NEA. Changes in TF activities can be measured from several perspectives: transcript abundance changes, post-translational modification changes of TF in cell signaling, TF-binding status to chromatin, relocalization status of the TF into/out of the nucleus, or changes in the abundance of TF’s target genes (Ma and Brent 2021, Weidemüller *et al.* 2021).

We compared the TF activity in transcriptome abundance level. As shown in Supplementary Fig. S7A, *CTNNB1* and *JUN* showed a non-significant difference between EA/NEA in transcriptome level (*t*-test *P* value after multiple test correction $5.85\times 10^{-2}$ and $7.30\times 10^{-2}$, respectively). Thus, DEG analysis can miss potentially important genes.

To overcome the limitation of DEG analysis, we further compared the activity of the TFs in two perspectives: (i) simulation of global effect of the TFs over all other genes via network propagation on PPI network, and (ii) the expression level changes of its direct target genes using gene regulatory network. Recent studies showed that network-based analysis can capture potentially important genes even when expression changes are minor. Since TF’s activity can be estimated via its target genes, we utilized network propagation to measure the effect of TFs although changes in TF’s expression are not significant.

#### 3.4.1 Network propagation analysis

We characterized genes that are most influenced by *CTNNB1* or *JUN*, which are the top 50 genes with the highest network propagation score. The propagation is conducted using *CTNNB1* or *JUN* as a seed gene on EA/NEA subtype-specific co-expression network, respectively (Supplementary method F). We found that subtype-specific genes are discovered, 27% of genes for *CTNNB1* and 47% of genes for *JUN* (Supplementary Fig. S7B). Then we conducted over-representation analysis of subtype-specific genes using GOBP database to show the biological function of subtype-specific genes. As shown in Supplementary Fig. S7C, EA-specific GOBP terms encompass functions such as the production of IL-4, a critical hallmark in the context of EA, known to induce airway eosinophilia and to promote bronchial hyper-responsiveness (Hoontrakoon *et al.* 1999, Kips *et al.* 2001). Meanwhile, in NEA subtype, notch receptor signaling is a well-known macrophage-regulating pathway (Palaga *et al.* 2008) and calcineurin-related signaling pathway. Calcineurin is a calcium/calmodulin-dependent serine/threonine protein phosphatase, which regulates T-cell activity (Rusnak and Mertz 2000). This implies that the genes affected by *CTNNB1* and *JUN* show distinct biological functions in each subtype.

#### 3.4.2 Transcriptional network analysis

Proteome abundance of JUN’s target genes showed a significant difference in expression level which clearly shows that the JUN’s activity differs between EA/NEA (Supplementary Fig. S7D, Supplementary method G). Among the nine target genes of *JUN* where proteome abundance is measured, four target genes showed significant differences in expression level between EA/NEA (*P* value < .05 after multiple test corrections). To demonstrate that *JUN*, not other TFs, yields protein-level difference of the target genes, we showed that *JUN* is the only TF shared by the target genes (Supplementary Fig. S7E). Since multiple studies have suggested that inferred TF activity level from changes in the expression levels of the TF’s target genes (Chen *et al.* 2017, Ma and Brent 2021), there exists possibility that TF activity of *JUN* is differentially modulated between EA/NEA via other TF activity regulating mechanisms, not by transcript level changes.

### 3.5 Discovery of network biomarkers that work cooperatively

Cooperative effects between biomarkers have been observed in heterogeneous diseases, where combinations of genes exhibit greater predictive power than any single gene in isolation(Sun *et al.* 2019). Biomarkers selected by GOAT showed high average node connectivity compared to the connectivity of genes selected by random sampling (permutation test *P* value = .001), which is exceptionally high compared to the biomarkers discovered by simple statistical analysis on single-omics data (Supplementary Fig. S8A, Supplementary method H). It implies that there exists dense interaction between the biomarkers which is crucial for cooperative function. Dropping performance in gene ablation study also suggests the existence of a cooperative effect (Supplementary Fig. S8B).

## 4 Discussion

The goal of our research is to identify biomarkers for asthma subtypes. Because asthma subtypes are not well-defined, we explored various subtype criteria by leveraging multi-omics explanatory power and we found that EA/NEA subtypes can be explained well in terms of molecular data.

To investigate the molecular mechanism of EA/NEA subtypes, we developed a deep-learning-based computational framework for biomarker discovery of EA/NEA subtypes by simultaneously considering multi-omics data. Based on the observation that there exists a hierarchy of multi-omics layers in explaining phenotypes, we used a biomarker prioritization procedure that utilizes proteome/metabolome as the origin of the flow of information to explain the phenotypes. To effectively consider complex interactions among genes, we first refined complex gene interactions by performing the network propagation analysis and then we developed a GNN-based biomarker identification method that models complex inter-omics and intra-omics relations with graph transformer. Note that the use of the techniques resulted in identifying biomarkers that are distinct only in terms of relationship in the multi-omics data. Thus, GOAT successfully discovered genes that can hardly be discovered by single-omics analysis. We also demonstrated that biomarkers discovered by GOAT showed high prediction performance for unseen patients and further showed that the biomarkers are likely to be regulated in a cooperative manner. However, some limitations remain, which should be addressed in the future to further improve the expandability of a model.

First, our approach for the integration of multi-omics data is based on the node features in GNN that represent multi-omics data without causal effects of multi-omics data. Indeed, existing methods (Stark *et al.* 2006, Szklarczyk *et al.* 2017) do not consider causal effects of multi-omics data. As graph learning technologies develop to a technical level of handling multi-layer networks effectively, GOAT may be revised accordingly to reflect the causal effects of multi-omics data.

Second, our approach, as most of existing state-of-the-art methods (Dimitrakopoulos *et al.* 2018, Jung *et al.* 2021), used a gene-centric approach for multi-omics integration. This strategy is reasonable since other genetic elements, i.e. epigenetic elements, control gene transcription. Computational models may have better predictive power for multi-omics biomarker discovery if simultaneous handling of multi-omics components is possible.

## Supplementary Material

btad582_Supplementary_Data
