# Supplementary material for: GOAT: Gene-level biomarker discovery from multi-Omics data using graph ATtention neural network for eosinophilic asthma subtype
Source: Bioinformatics. 2023 Sep 22;39(10):btad582. doi: 10.1093/bioinformatics/btad582 (PMC10547929; doi:10.1093/bioinformatics/btad582)
Supplement: btad582_Supplementary_Data [file btad582_supplementary_data.pdf]

Supplementary tables

Table S1: **Subtype criterion** Asthma subtypes classified by clinicians in Asan medical center.

Subtype code	Criterion	Subtype class
SUB_ACO	Asthma-COPD overlap	**1**: yes / **2**: no or NA
SUB_FEV1%	Percentage of forced expiratory volume in one second	**1**: ratio < 60 / **2**: $60 \leq$ ratio < 80 / **3**: $80 \leq$ ratio
SUB_FEV1/FVC	FEV1/FVC ratio	**1**: ratio < 0.7 / **2**: $0.7 \leq$ ratio
SUB_ICS_DOSE	Inhaled corticosteroid dose	**1**: medium or high / **2**: low
SUB_ACT	Total score of Asthma Control Test (ACT)	**1**: score ≤ 19 / **2**: $20 \leq$ score
SUB_CONTROL	Asthma control status	**1**: controlled / **2**: partially controlled / **3**: uncontrolled
SUB_EXAC_EVER	The number of exacerbation visits ever	**1**: $1 \leq$ # visits / **2**: # visits = 0 or NA
SUB_EXAC_1Y	The number of exacerbation visits in a year	**1**: $1 \leq$ # visits / **2**: # visits = 0 or NA
PH_EOSI_B_150	Blood eosinophil count (BEC) < 150 μmol$/l$	**1**: counts < 150 / **2**: $150 \leq$ counts
PH_EOSI_B_300	Blood eosinophil count (BEC) < 300 μmol$/l$	**1**: counts < 300 / **2**: $300 \leq$ counts
PH_EOSI_S	Sputum eosinophil count (SEC)	**1**: counts < 2 / **2**: $2 \leq$ counts
PH_FENO_25	Fractional exhaled nitric oxide (FeNO) test score < 25	**1**: score < 25 / **2**: $25 \leq$ score
PH_FENO_50	Fractional exhaled nitric oxide (FeNO) test score < 50	**1**: score < 50 / **2**: $50 \leq$ score
PH_ATOPY	Atopy	**1**: non-atopic / **2**: atopic
PH_TOIGE	Total IgE level	**1**: level < 100 / **2**: $100 \leq$ level
PH_T2	T2 low/high	**1**: (BEC<300 and nonatopic) and (FeNO<25 or SEC<3)/ **2**: the other

Table S2: **Over-representation analysis of gene modules connected via *CTNNB1*** Enriched gene ontology biological process (GOBP) terms with gene modules connected to *CTNNB1*. Over-representation analysis is conducted with EnrichR (Kuleshov et al., 2016)

GOBP term	Adj. P-value
Negative regulation of apoptotic process	2.108e-13
Platelet degranulation	1.625e-12
Cellular protein modification process	3.305e012
Regulated exocytosis	3.768e-12
Positive regulation of DNA-binding transcription factor activity	2.258e-10

Table S3: **Over-representation analysis of gene modules connected via *JUN*** Enriched gene ontology biological process (GOBP) terms with gene modules connected to *JUN*. Over-representation analysis is conducted with EnrichR (Kuleshov et al., 2016)

GOBP term	Adj. P-value
MAPK cascade	1.327e-13
Negative regulation of apoptotic process	2.865e-11
Stress-activated protein kinase signaling cascade	3.944e-10
Positive regulation of protein localization to membrane	3.944e-10
Negative regulation of programmed cell death	6.685e-10
Response to host defenses	1.670e-08
Modulation of symbiont of host defense response	1.670e-08
Regulation of apoptotic process	2.967e-08

Table S4: **TF enrichment** TF enrichment analysis of the biomarkers discovered via EnrichR (Chen et al., 2013) based on TRRUST_Transcription_Factors_2019 databse.

Gene	Overlap	adj. P value	Overlapped genes
JUN	8/149	0.000732	*APP*;*LDHA*;*JUN*;*IL6*;*MAPK8*;*MYC*;*TP53*;*EGFR*
CTNNB1	3/22	0.007404	*JUN*;*MYC*;*PLD1*

Supplementary figures

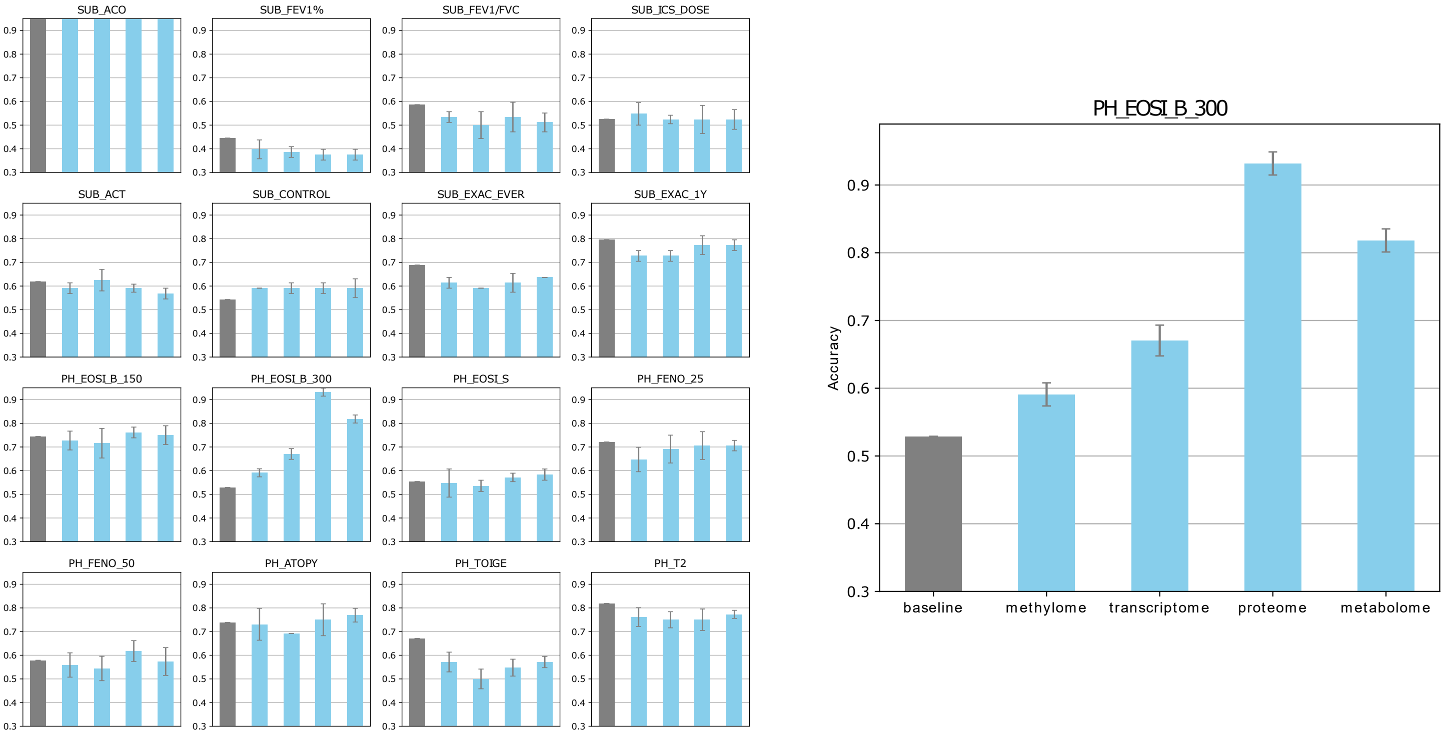

Fig. S1: **Eosinophilic asthma/non-eosinophilic asthma is the asthma subtype that is discriminable with multi-omics data** Each bar plot shows the prediction accuracy of each subtype defined in Table S1. The right panel shows the bar plot of Eosinophilic asthma (EA) and non-eosinophilic asthma (NEA). Blue bars depict the 10-fold cross-validation prediction accuracy from random forest classifier using single omics data—methylome, transcriptome, proteome, and metabolome. On the contrary, the grey bar depicts baseline prediction accuracy which is the ratio of the majority subtype class over total samples. The more imbalanced the subtype class is, the higher the baseline prediction accuracy is. We compared the prediction accuracy of each omics data compared to the baseline. Our goal is to determine subtypes that show high prediction accuracy due to the explanatory power of each omics, not by the random guessing of subtype class to the majority class.

Gene symbol	Full name	Is Secretory protein?		Is Cytoskeletal protein?
		(Secretome DB: Protein Atlas)	(Subcellular locations from UniProtKB/Swiss-Prot: "Secreted" from Genecards)	(Subcellular locations from UniProtKB/Swiss-Prot: "Cytoskeleton" from Genecards)
A2M	Alpha-2-macroglobulin	O	O	
ALB	Serum albumin	O	O	
APOL1	Apolipoprotein L1	O	O	
HP	Haptoglobin-related protein	O	O	
IGF2R	Cation-independent mannose-6-phosphate receptor	△ (Counterpart to secretory protein Insulin like growth factor 2, IGF-2)		
IGKC	Immunoglobulin Kappa			
MYH9	Myosin-9			O
TNXB	Tenascin-X (extracellular matrix glycoproteins regulating the rigidity or elasticity of virtually every cell in the body)		O	
TPM3	Tropomyosin alpha-3 chain			O

Fig. S2: **Important genes discovered solely with proteome is secretory/structural proteins** List of proteins discovered as important features in random forest model using proteome data. It is the gene-level inspection of the results of proteome from Fig.S1. Feature importance is computed as permutation importance for 5 repeats using *permutation_importance* method from sklearn.inspection module (Breiman, 2001). The proteins are selected as important features when the mean importance over the repeats is non-zero. The function of each protein is annotated by the Secretome database of Protein Atlas (Uhlén et al., 2019) and GeneCards (Stelzer et al., 2016).

A.

	AUPRC				AUROC			
	Median		IQR		median		IQR	
DEG	0.69	+36.2%	0.029	+58.6%	0.69	+34.7%	0.026	+26.9%
Multi-omics NP	0.94		0.012		0.93		0.019	
DEP	0.93	+1.1%	0.018	+33.3%	0.92	+1.1%	0.026	+26.9%
Multi-omics NP	0.94		0.012		0.93		0.019	

B.

	AUROC				AUPRC			
	median		IQR		median		IQR	
SVM	0.84	+10.7%	0.044	+56.8%	0.89	+5.62%	0.027	+55.6%
GNN	0.93		0.019		0.94		0.012	
XGBoost	0.91	+2.2%	0.024	+20.8%	0.92	+2.2%	0.045	+73.3%
GNN	0.93		0.019		0.94		0.012	
MLP	0.85	+9.4%	0.021	+9.5%	0.82	+14.6%	0.051	+76.5%
GNN	0.93		0.019		0.94		0.012	

Fig. S3: **Subtype prediction performance evaluation** Table comparing the AUPRC/AUROC of **(A)** the network propagation based multi-omics feature selection method (multi-omics NP) vs. single-omics feature selection method and **(B)** the GNN classifiers versus other classifiers. AUPRC, Area under the precision-recall curve; AUROC, Area under the receiver operating characteristic curve; IQR, Inter Quartile Range; NP, Network propagation; DEG, Differentially expressed gene; DEP, Differentially expressed protein; SVM, Support vector machine; GNN, Graph neural network; MLP; Multi-layer perceptron.

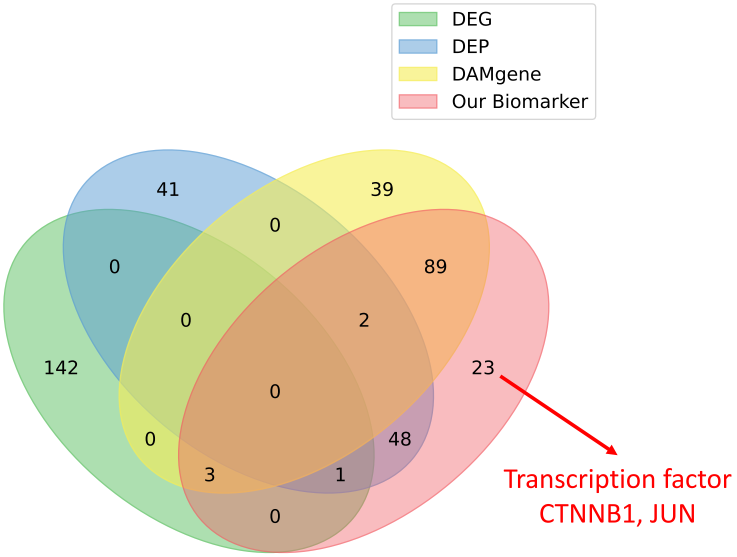

Fig. S4: **Venn diagram for genes discovered by single omics analysis versus GOAT** Using single omics data, differentially expressed genes are discovered via simple statistical method for transcriptome, proteome (Supplementary method B), or DAMgenes discovered via metPropgate (Graham Linck et al., 2020). Among the genes solely discovered by GOAT, there exist transcription factors, *CTNNNB1* and *JUN*.

A.

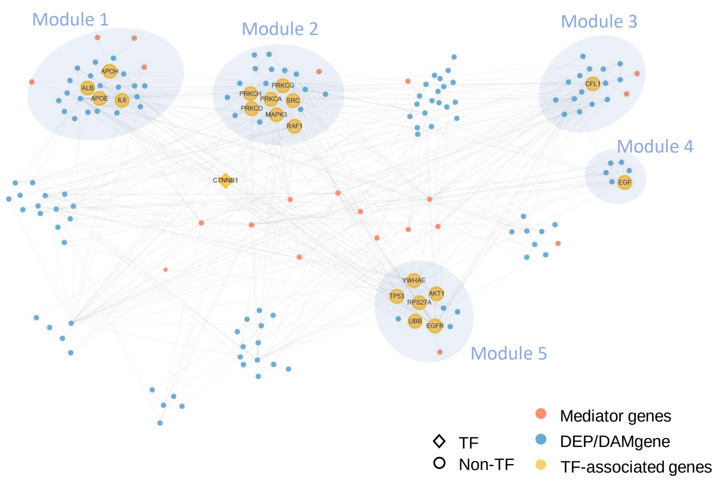

B.

Module 1

Name	Adjusted p-value
triglyceride-rich lipoprotein particle remodeling (GO:0034370)	1.443e-9
cellular protein metabolic process (GO:0044267)	7.460e-9
positive regulation of lipid metabolic process (GO:0045834)	1.763e-8
platelet degranulation (GO:0002576)	1.763e-8
positive regulation of cholesterol esterification (GO:0010873)	1.770e-8

Module 2

Name	Adjusted p-value
polyol metabolic process (GO:0019751)	3.702e-14
inositol phosphate metabolic process (GO:0043647)	3.702e-14
phosphate-containing compound metabolic process (GO:0006796)	9.616e-13
Fc-gamma receptor signaling pathway involved in phagocytosis (GO:0038096)	8.120e-11
Fc-gamma receptor signaling pathway (GO:0038094)	8.120e-11

Module 3

Name	Adjusted p-value
positive regulation of protein localization to early endosome (GO:1902966)	2.610e-6
positive regulation of protein localization to endosome (GO:1905668)	2.610e-6
regulation of protein localization to early endosome (GO:1902965)	2.610e-6
regulation of cellular component biogenesis (GO:0044087)	2.610e-6
interleukin-12-mediated signaling pathway (GO:0035722)	3.858e-6

Module 4

Name	Adjusted p-value
platelet degranulation (GO:0002576)	1.304e-11
regulated exocytosis (GO:0045055)	6.036e-11
positive regulation of phosphate metabolic process (GO:0045937)	2.145e-3
peptide cross-linking (GO:0018149)	2.145e-3
regulation of cellular component movement (GO:0051270)	4.510e-3

Module5

Name	Adjusted p-value
stress-activated protein kinase signaling cascade (GO:0031098)	1.567e-7
negative regulation of apoptotic process (GO:0043066)	1.567e-7
response to host defenses (GO:0052200)	3.587e-7
modulation by symbiont of host defense response (GO:0052031)	3.587e-7
MAPK cascade (GO:0000165)	3.587e-7

Fig. S5: **Subnetwork of biomarkers and functional analysis of gene modules, connected to *CTNNB1* (A)** Gene modules from network biomarkers. Each node in a network denotes a gene, and the edge is retrieved from the gene-interaction network exploited in graph neural network model. Rhombus indicates transcription factors while the circle indicates non-TFs. Blue nodes are functional genes that can be discovered by proteome or metabolome analysis, denoted DEP / DAM genes. Gene modules of functional genes are clustered via GLay algorithm (Su et al., 2010). Orange nodes are the genes solely by GOAT that are connected to the gene modules, denoted as mediator genes. Yellow nodes are the genes connected to *CTNNB1*. Blue shade indicates functional modules connected to *CTNNB1*. **(B)** Enriched gene ontology biological process terms of each module. TF, Transcription factor; DEP, Differentially expressed protein; DAMgene, Differentially abundant metabolite (DAM)-related gene.

A.

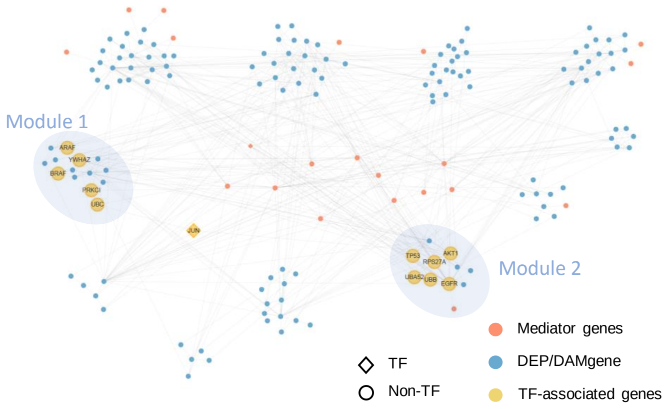

B.

Module 1

Name	Adjusted p-value
MAPK cascade (GO:0000165)	9.080e-6
phosphorylation (GO:0016310)	2.352e-5
positive regulation of protein localization to membrane (GO:1905477)	5.721e-5
regulation of MAPK cascade (GO:0043408)	2.499e-4
negative regulation of programmed cell death (GO:0043069)	2.499e-4

Module 2

Name	Adjusted p-value
stress-activated protein kinase signaling cascade (GO:0031098)	1.567e-7
negative regulation of apoptotic process (GO:0043066)	1.567e-7
response to host defenses (GO:0052200)	3.587e-7
modulation by symbiont of host defense response (GO:0052031)	3.587e-7
MAPK cascade (GO:0000165)	3.587e-7

Fig. S6: **Subnetwork of biomarkers and functional analysis of gene modules, connected to *JUN* (A)** Gene modules from network biomarkers. Each node in a network denotes a gene and the edge is retrieved from the gene-interaction network exploited in graph neural network model. Rhombus indicates transcription factors (TFs) while the circle indicates non-TFs. Blue nodes are functional genes that can be discovered by proteome or metabolome analysis, denoted DEP / DAM genes. Gene modules of functional genes are clustered via GLay algorithm (Su et al., 2010). Orange nodes are the genes solely by GOAT that are connected to the gene modules, denoted as mediator genes. Yellow nodes are the genes connected to *JUN*. Blue shade indicates functional modules connected to *JUN*. **(B)** Enriched gene ontology biological process terms of each module. TF, Transcription factor; DEP, Differentially expressed protein; DAMgene, Differentially abundant metabolite (DAM)-related gene.

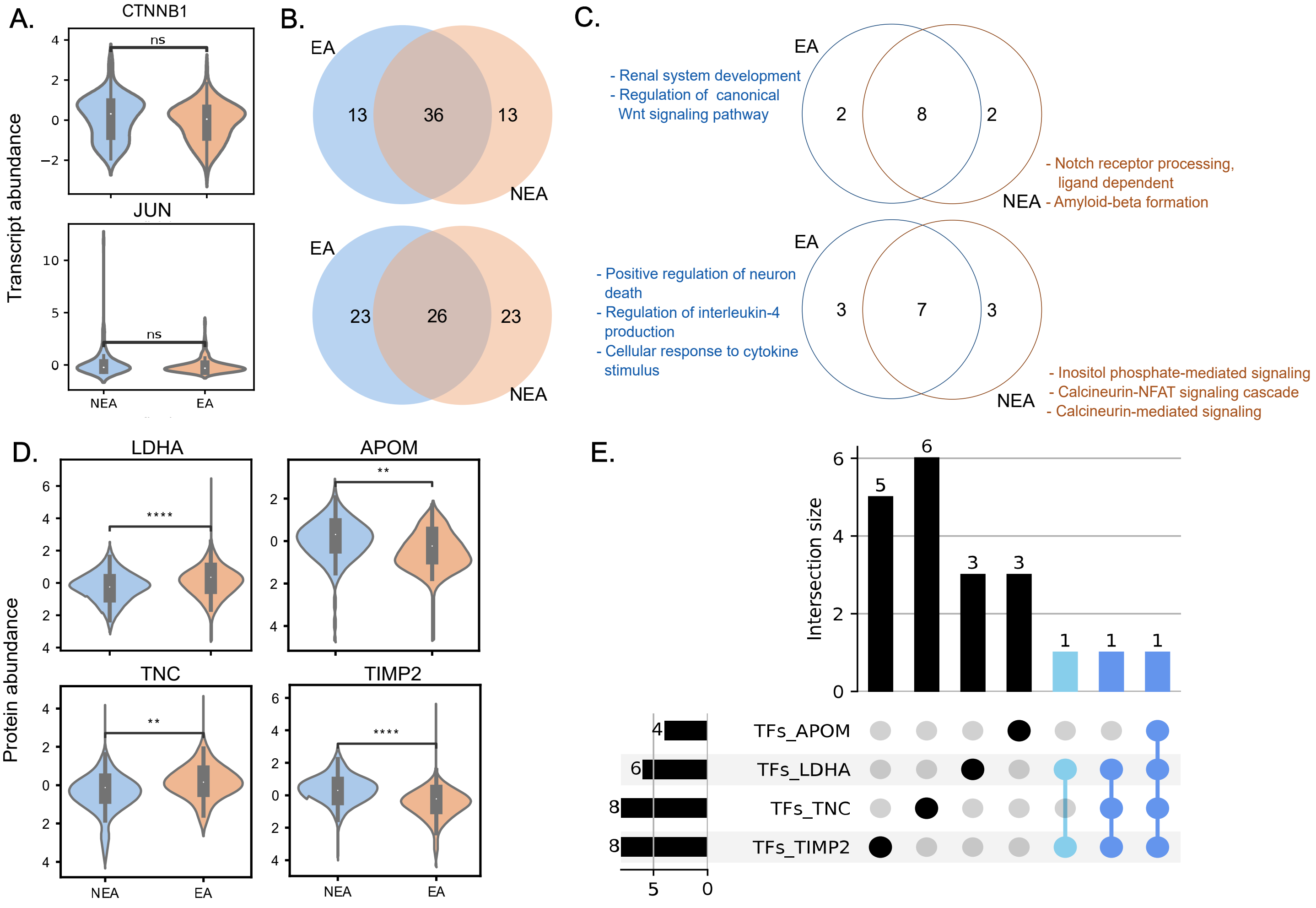

Fig. S7: ***CTNNB1/JUN*-related genes show distinct pattern in Eosinophilic asthma vs. Non-eosinophilic asthma** **(A)** Violin plot of gene expression of *CTNNNB1* and *JUN* in transcriptome level comparing Eosinophilic asthma (EA) and Non-eosinophilic asthma (NEA). **(B)** Venn diagram of the genes most affected by *CTNNNB1* and *JUN*, comparing EA/NEA (Detailed descriptions are in the Supplementary method F). **(C)** Venn diagram of biological process terms of the gene ontology enriched with subtype-specific genes discovered in B. **(D)** Violin plot of gene expression of *JUN*'s target gene (TG) in proteome level comparing EA/NEA. **(E)** Upset plot to display all intersections of TF sets for each TG in D, sorted by size. TFs_{*gene*} indicates the set of TFs for the *gene* retrieved from TRRUST database (Detailed descriptions are in Supplementary method G). Dark circles indicate sets that are part of the intersection, and horizontal bars indicate the size of each TF set. Blue shade indicates intersection shared by multiple TF sets. Deep blue shade indicates the intersection of TFs shared by all target genes. (Statistical annotations with t-test, ns: non-significant, **: $p<0.01$, ****: $p<0.0001$). TF, Transcription factor; EA, Eosinophilic asthma; NEA, Non-eosinophilic asthma.

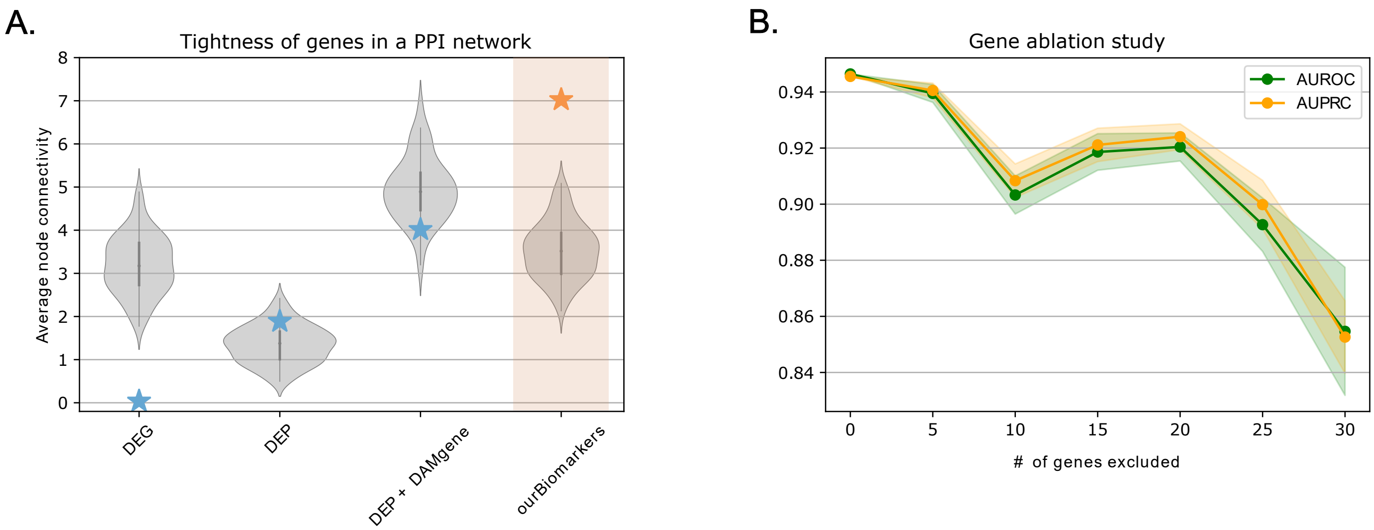

Fig. S8: **Existence of cooperative effect of biomarkers (A)** Average node connectivity of genes in a protein-protein interaction network comparing the biomarkers identified by GOAT with other biomarker discovery methods based on statistical testing (Detail in Supplementary method H). Violin plots depict the null distribution of average node connectivity. The star denotes the test statistic, the average node connectivity of the specified biomarker set discovered with the methods in the x-axis. **(B)** Line plot of test AUPRC/AUROC decaying according to the number of genes excluded. To exclude the effect of the randomly selected genes in a model, all selected features were set to zero. For exclusion of the specified number of genes, random exclusion is repeated 10 times so that the interquartile range is depicted as shades. DEG, Differentially expressed gene; DEP, Differentially expressed protein; DAMgene, Differentially abundant metabolite (DAM)-related gene; AUPRC, Area under the precision-recall curve; AUROC, Area under the receiver operating characteristic curve.

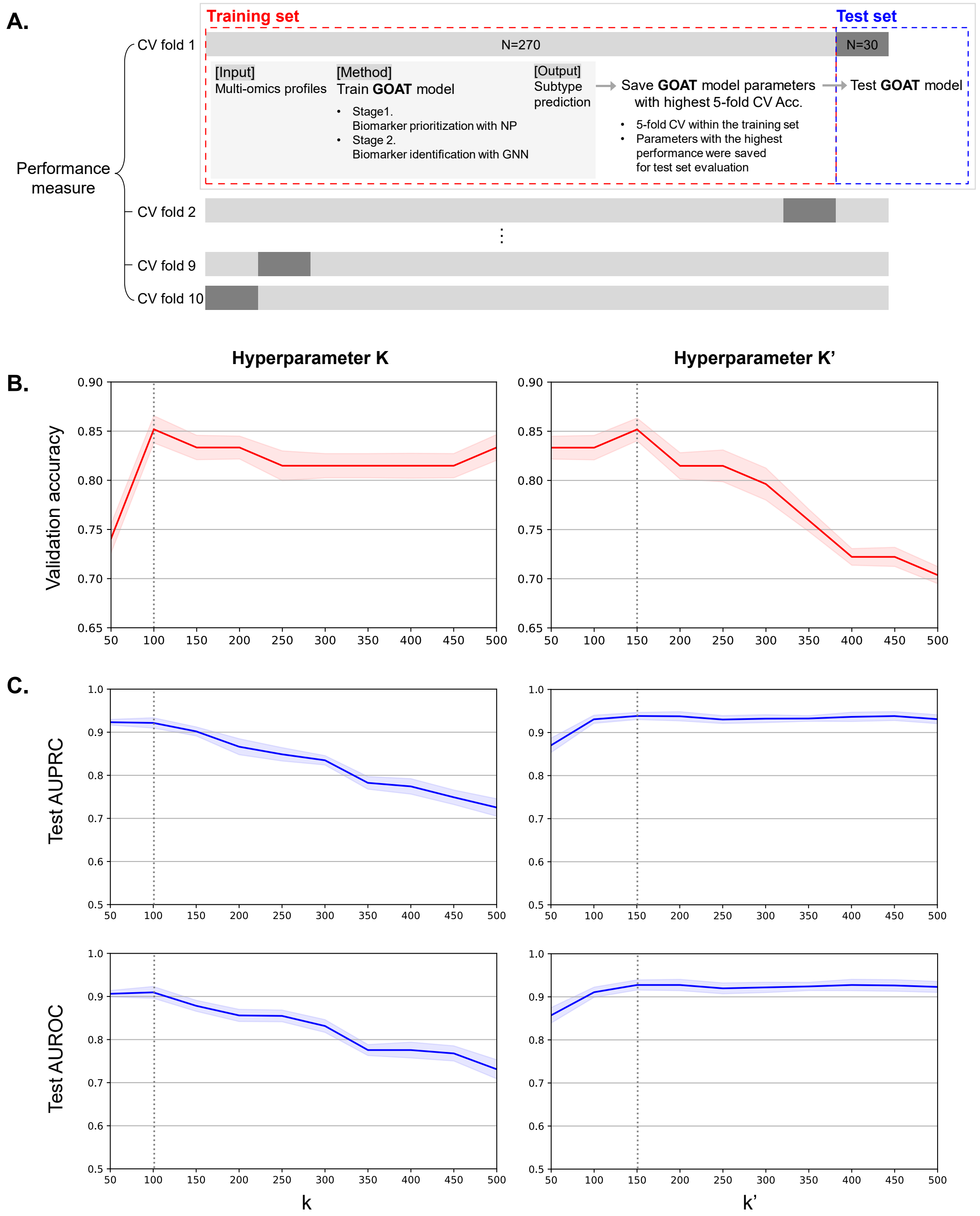

Fig. S9: **Hyperparameter search for biomarker candidate prioritization (Stage 1) (A)** Cross-validation scheme for hyperparameter search. 5-fold CV was conducted within training set, where each fold was set as held-out set for validation and other folds were used for model training. Median of prediction accuracy across 5 CV-folds, called validation accuracy, was measured for hyperparameter tuning. **(B)** Line plot of validation accuracy with increasing k, k'. Red line in the plots depicts validation accuracy and light red shade depicts standard deviation of validation accuracy over 10-fold CV. Hyperparameter k signifies the number of DAMgenes from met-Propagate(Graham Linck et al., 2020) to be screened for the following procedures in stage 1 of GOAT and k' signifies the number of biomarker candidate genes from network propagation to be screened for the following procedures in stage 2 of GOAT. k, k' that showed best prediction performance within validation set are selected for test set evaluation. **(C)** Line plot of test AUPRC/AUROC with increasing k, k'. Blue line in the plots depicts test AUPRC/AUROC and light blue shade depicts standard deviation of test AUPRC/AUROC over 10-fold CV. CV, Cross-validation; Acc, Accuracy; AUPRC, Area under the precision-recall curve; AUROC, Area under the receiver operating characteristic curve; DAMgene, Differentially abundant metabolite (DAM)-related gene.

Supplementary methods

A Exploration of asthma subtypes in terms of multi-omics data

We explored several physiological/clinical subtype criteria because the criterion can be explainable with multi-omics data. From the subtypes defined according to the 16 phenotypic or endotypic features in COREA asthma cohort (TableS1), eosinophilic asthma (EA, Blood eosinophil count ≥ 300) and non-eosinophilic asthma (NEA, Blood eosinophil count < 300) is the subtype that shows comparable discriminative power in each omics data compared to the random prediction of subtypes, while none of the omics data showed comparable discriminative power according to the remaining subtype criteria (Fig.S1 left panel).

We explored the discriminative power of each omics in subtype classification to gain a picture of how multiple omics layers are related. We used random forest classifier (Breiman, 2001) for each omics data and retrieved feature importance score of omics features. To this end, we discovered that proteome and metabolome showed the best discriminative power of 0.93 accuracy and 0.82 accuracy, respectively, much better than methylome and transcriptome (Fig. S1 right panel). However, the genes that show high feature importance in proteome analysis are restricted to protein-coding genes that encode secretory or cytoskeletal proteins, which are effector/reactor, not regulatory genes (Fig.S2). To explain the pathophysiology of the subtype, both effector genes and regulatory genes need to be identified. Thus it is important to discover regulatory genes and their regulatory network of genes that explain the difference between EA/NEA subtypes, starting from metabolome and proteome that include effector genes.

B Differentially expressed omics (DEomics) detection

Differentially abundant features of each omics are computed with the nonparametric statistical test, Wilcoxon rank sum test (Mann and Whitney, 1947). For each omics, we filter the features with a significance level of 0.05 after multiple test corrections with Sidak adjustments (Šidák, 1967). Subtype-specific differentially abundant metabolites (DAMs) and differentially expressed proteins (DEPs) are defined as the up-regulated DAMs/DEPs in one subtype but not in the other; i.e. genes with adjusted *P* value < 0.05 and Wilcoxon rank-sum test statistic > 0 are regarded as subtype-specific DEP/DAM for one subtype and adjusted *P* value < 0.05 and Wilcoxon rank-sum test statistic < 0 are regarded as subtype-specific DEP/DAM for the other.

C Gene-gene interaction network construction

C.1 Gene-gene interaction network

We used a union of STRING (v10.5) (Szklarczyk et al., 2017) and BioGrid (v.3.5.179) (Stark et al., 2006) database as a gene-gene interaction network. Since STRING provides a confidence score for each edge, gene-gene interaction with a confidence score < 0.8 is filtered out for STRING. We then constructed a graph consisting of a union of STRING and BioGrid edges to be used as an adjacency matrix W_i.

C.2 Gene functional similarity network

We used the GOBP database (Consortium, 2021) to calculate the functional similarity of gene pairs. The database is transformed as a graph of gene ontology (GO) through the Knowledge Graph Hub platform, Knowledge Graph Hub (KG Hub).

We assumed that genes that share GO terms with high semantic similarity in a GO tree show high functional similarity. Semantic similarity is a metric to measure the similarity of two concepts organized in an ontology (Couto et al., 2007). For a rooted tree T consisting of GO terms where the root is r, the semantic similarity between two GO term nodes u, v in a tree is computed as follows;

$$\text{sim}(u, v) = \frac{1}{1 + d_s(u, v)}$$
$$d_s(u, v) = d_T(r, u) + d_T(r, v) - 2d_T(r, \text{lca}(u, v))$$

where $\mathrm{d}_T(\cdot)$ is shortest path length and $\text{lca}(\cdot)$ is the lowest common ancestor between nodes. The average semantic similarity of all possible combinations of GO terms between genes is computed to construct the functional similarity graph to be utilized as a weighted adjacency matrix W_f.

D Data preprocessing

The multi-omics dataset of COREA cohort is downloaded from Multi-Omics Platform (`http://203.252.206.90:5566/`). Raw data can be accessible when requested. Methylation data is generated by bisulfite-sequencing and aligned to hg19 genome. Methylation status is measured in 3,419,515 CpG sites and transformed into gene-level methylation status by averaging methylation level of CpG sites within ± 2kb on transcription start site (TSS), using TSS annotation of hg19 from UCSC Genome Browser (Navarro Gonzalez et al., 2021). Transcriptome data is generated by RNA-seq using HISAT2 (Kim et al., 2015) (v2.2.0) and processed with samtools (Li et al., 2009) (v1.9) and HTSeq (Anders et al., 2015) (v0.12.4), where ucsc hg19 genome is utilized as a reference for each step. Raw read counts are normalized to counts per million. Proteome data is generated by SWATH mass spectrometry (MS), processed as a peak intensity file with Perseus (Tyanova et al., 2016) (v1.6.5), and processed as in protein-level abundance with R DEP package (Zhang et al., 2018). Metabolome data is generated via targeted liquid chromatography-MS of 400 endogenous metabolites and processed as a metabolite abundance with MetIDQ from Biocrates. Clinical subtypes are defined by the following clinical features (Table S1).

E Training and validation strategy

E.1 Cross-validation scheme

We performed 10-fold cross-validation scheme where each set (N=30) became a held-out test set, while the other nine sets (N=270) were used for both biomarker prioritization (Stage 1) and biomarker identification (Stage 2) (Fig.S9A). Identical split of test set is fed into all methods in comparative analysis, including ours. To ensure there is no information leak, only samples from the training set were used to prioritize biomarkers by optimizing the network propagation parameters (stage 1) of our biomarker identification model (stage 2).

E.2 Parameter optimization

In the first stage of prioritization of biomarker candidates, hyperparameters are k was set to 100, α was set to 0.8, β was set to 0.5, and $k^{'}$ was set to 150. For hyperparameter tuning, prediction performance was assessed as 5-fold cross-validation accuracy within the training set, which is called validation accuracy. Parameters with the highest prediction performance for validation sets were saved for test set evaluation (Fig.S9B). For the hyperparameter sensitivity analysis shown in Fig.S9C, the prediction performance for the test set gradually decreases as k increases and the prediction performance becomes saturated as $k^{'}$ increases. We reported the test performance reported in Fig.2 with the best combination of hyperparameters selected using validation accuracy, $k = 100$ and $k^{'} = 150$.

In the second stage of identification of biomarkers, parameters consist of model parameter to be optimized during training process and hyperparameter to be designated outside of the model. For both model parameter optimization and hyperparameter tuning, prediction performance was evaluated as 5-fold cross-validation accuracy within training set and parameters with the highest performance were saved for test set evaluation (Fig.S9A). We used Adam Optimizer to optimize the model parameters. The hyperparameters were tuned by grid search, as a combination of learning rate [0.1, 0.01, 0.001, 0.0001], dropout rate [0.1, 0.2, 0.3, 0.4, 0.5], and feature dimension of node embeddings in graph neural network [4, 8, 16, 32, 64, 128]. Each combination of hyperparameters was fed into the model and evaluated as 5-fold cross-validation accuracy. Selected hyperparameters are 0.001 for learning rate, 0.2 for dropout rate, and 8 for feature dimension of node embeddings.

F Network propagation analysis

The specific network for the eosinophilic asthma / non-eosinophilic asthma subtype is constructed as a coexpression network of transcriptome data using samples of each subtype. Given a protein-protein interaction (PPI) network as a template, we computed edge weight as a Pearson correlation of each pair of genes in a PPI network. Edge weight higher than 0.3 is rescued to construct subtype-specific networks, $\mathbf{W}$. For each transcription factor (TF), the node attribute of the selected TF is set to 1 and the resource of the node is propagated to all other nodes in a network according to the following equation,

$$\mathbf{p}_t = (1-\alpha)\mathbf{W}\mathbf{p}_{t-1} + \alpha\mathbf{p}_0$$

G Transcriptional network analysis

TRRUST is a database of gene regulatory networks whose interactions are retrieved from literature mining (Han et al., 2018). For the TFs, *CTNNB1* and *JUN*, we trimmed downstream target genes of the TFs from TRRUST. The omic profile of the downstream target genes from the COREA cohort is examined.

H Biomarkers cooperativity

The tightness of genes in G in a protein-protein interaction network is defined as follows,

$$\kappa(G) = \frac{\sum_{u,v} \kappa_G(u,v)}{\binom{n}{2}}$$

where $\kappa_G(\cdot)$ is the minimum number of nodes that must be removed to disconnect two non-adjacent nodes. We inferred null distribution with random sampling of $|G|$ genes 100 times. Permutation test *P* value is computed according to the null distribution of a test statistic $\kappa(G)$ of the specified genes.

References

Simon Anders, Paul Theodor Pyl, and Wolfgang Huber. Htseq—a python framework to work with high-throughput sequencing data. *Bioinform.*, 31(2):166–169, 2015.

Leo Breiman. Random forests. *Machine learning*, 45(1):5–32, 2001.

Edward Y Chen, Christopher M Tan, Yan Kou, Qiaonan Duan, Zichen Wang, Gabriela Vaz Meirelles, Neil R Clark, and Avi Ma'ayan. Enrichr: interactive and collaborative html5 gene list enrichment analysis tool. *BMC Bioinform.*, 14(1):1–14, 2013.

Gene Ontology Consortium. The gene ontology resource: enriching a gold mine. *Nucleic Acids Res.*, 49(D1):D325–D334, 2021.

Francisco M Couto, Mario J Silva, and Pedro M Coutinho. Measuring semantic similarity between gene ontology terms. *Data & knowledge engineering*, 61(1):137–152, 2007.

Emma J Graham Linck, Phillip A Richmond, Maja Tarailo-Graovac, Udo Engelke, Leo AJ Kluijtmans, Karlien LM Coene, Ron A Wevers, Wyeth Wasserman, Clara DM van Karnebeek, and Sara Mostafavi. metpropagate: network-guided propagation of metabolomic information for prioritization of metabolic disease genes. *NPJ Genom. Med.*, 5(1):1–11, 2020.

Heonjong Han, Jae-Won Cho, Sangyoung Lee, Ayoung Yun, Hyojin Kim, Dasom Bae, Sunmo Yang, Chan Yeong Kim, Muyoung Lee, Eunbeen Kim, et al. Trrust v2: an expanded reference database of human and mouse transcriptional regulatory interactions. *Nucleic Acids Res.*, 46(D1):D380–D386, 2018.

Daehwan Kim, Ben Langmead, and Steven L Salzberg. Hisat: a fast spliced aligner with low memory requirements. *Nat. methods*, 12 (4):357–360, 2015.

Maxim V Kuleshov, Matthew R Jones, Andrew D Rouillard, Nicolas F Fernandez, Qiaonan Duan, Zichen Wang, Simon Koplev, Sherry L Jenkins, Kathleen M Jagodnik, Alexander Lachmann, et al. Enrichr: a comprehensive gene set enrichment analysis web server 2016 update. *Nucleic acids research*, 44(W1):W90–W97, 2016.

Heng Li, Bob Handsaker, Alec Wysoker, Tim Fennell, Jue Ruan, Nils Homer, Gabor Marth, Goncalo Abecasis, and Richard Durbin. The sequence alignment/map format and samtools. *Bioinform.*, 25(16):2078–2079, 2009.

Henry B Mann and Donald R Whitney. On a test of whether one of two random variables is stochastically larger than the other. *The annals of mathematical statistics*, pages 50–60, 1947.

Jairo Navarro Gonzalez, Ann S Zweig, Matthew L Speir, Daniel Schmelter, Kate R Rosenbloom, Brian J Raney, Conner C Powell, Luis R Nassar, Nathan D Maulding, Christopher M Lee, et al. The ucsc genome browser database: 2021 update. *Nucleic Acids Res.*, 49(D1):D1046–D1057, 2021.

Chris Stark, Bobby-Joe Breitkreutz, Teresa Reguly, Lorrie Boucher, Ashton Breitkreutz, and Mike Tyers. Biogrid: a general repository for interaction datasets. *Nucleic Acids Res.*, 34(suppl_1):D535–D539, 2006.

Gil Stelzer, Naomi Rosen, Inbar Plaschkes, Shahar Zimmerman, Michal Twik, Simon Fishilevich, Tsippi Iny Stein, Ron Nudel, Iris Lieder, Yaron Mazor, et al. The genecards suite: from gene data mining to disease genome sequence analyses. *Current protocols in Bioinform.*, 54(1):1–30, 2016.

Gang Su, Allan Kuchinsky, John H Morris, David J States, and Fan Meng. Glay: community structure analysis of biological networks. *Bioinform.*, 26(24):3135–3137, 2010.

D Szklarczyk, JH Morris, H Cook, M Kuhn, S Wyder, M Simonovic, and A Santos. The string database in 2017: quality-controlled protein–protein association networks, made broadly accessible. *Nucleic Acids Res.*, 45:D362–d368, 2017.

Stefka Tyanova, Tikira Temu, Pavel Sinitcyn, Arthur Carlson, Marco Y Hein, Tamar Geiger, Matthias Mann, and Jürgen Cox. The perseus computational platform for comprehensive analysis of (prote) omics data. *Nat. Methods*, 13(9):731–740, 2016.

Mathias Uhlén, Max J Karlsson, Andreas Hober, Anne-Sophie Svensson, Julia Scheffel, David Kotol, Wen Zhong, Abdellah Tebani, Linnéa Strandberg, Fredrik Edfors, et al. The human secretome. *Science signaling*, 12(609):eaaz0274, 2019.

Xiaofei Zhang, Arne H Smits, Gabrielle van Tilburg, Huib Ovaa, Wolfgang Huber, and Michiel Vermeulen. Proteome-wide identification of ubiquitin interactions using ubia-ms. *Nat. Protocols*, 13(3):530–550, 2018.

Zbyněk Šidák. Rectangular confidence regions for the means of multivariate normal distributions. *Journal of the American Statistical Association*, 62(318):626–633, 1967. doi: 10.1080/01621459.1967.10482935.
